# NSC19723, a Thiacetazone-Like Benzaldehyde Thiosemicarbazone Improves the Efficacy of TB Drugs *In Vitro* and *In Vivo*

**DOI:** 10.1128/spectrum.02592-22

**Published:** 2022-10-31

**Authors:** Padam Singh, Srishti Rawat, Ashish Kumar Agrahari, Manisha Singh, Saurabh Chugh, Sudagar Gurcha, Albel Singh, Katherine Abrahams, Gurdyal S. Besra, Shailendra Asthana, Diwan S. Rawat, Ramandeep Singh

**Affiliations:** a Translational Health Science and Technology Institute, NCR Biotech Science Cluster, Faridabad, Haryana, India; b Department of Chemistry, University of Delhi, Delhi, India; c Institute of Microbiology and Infection, School of Biosciences, University of Birmingham, Birmingham, United Kingdom; Emory University School of Medicine

**Keywords:** *Mycobacterium tuberculosis*, NSC19723, HadABC, synergy, thiacetazone

## Abstract

The complexity and duration of tuberculosis (TB) treatment contributes to the emergence of drug resistant tuberculosis (DR-TB) and drug-associated side effects. Alternate chemotherapeutic agents are needed to shorten the time and improve efficacy of current treatment. In this study, we have assessed the antitubercular activity of NSC19723, a benzaldehyde thiosemicarbazone molecule. NSC19723 is structurally similar to thiacetazone (TAC), a second-line anti-TB drug used to treat individuals with DR-TB. NSC19723 displayed better MIC values than TAC against Mycobacterium tuberculosis and Mycobacterium bovis BCG. In our checkerboard experiments, NSC19723 displayed better profiles than TAC in combination with known first-line and recently approved drugs. Mechanistic studies revealed that NSC19723 inhibits mycolic acid biosynthesis by targeting the HadABC complex. Computational studies revealed that the binding pocket of HadAB is similarly occupied by NSC19723 and TAC. NSC19723 also improved the efficacy of isoniazid in macrophages and mouse models of infection. Cumulatively, we have identified a benzaldehyde thiosemicarbazone scaffold that improved the activity of TB drugs in liquid cultures, macrophages, and mice.

**IMPORTANCE** Mycobacterium tuberculosis, the causative agent of TB is among the leading causes of death among infectious diseases in humans. This situation has worsened due to the failure of BCG vaccines and the increased number of cases with HIV-TB coinfections and drug-resistant strains. Another challenge in the field is the lengthy duration of therapy for drug-sensitive and -resistant TB. Here, we have deciphered the mechanism of action of NSC19723, benzaldehyde thiosemicarbazone. We show that NSC19723 targets HadABC complex and inhibits mycolic acid biosynthesis. We also show that NSC19723 enhances the activity of known drugs in liquid cultures, macrophages, and mice. We have also performed molecular docking studies to identify the interacting residues of HadAB with NSC19723. Taken together, we demonstrate that NSC19723, a benzaldehyde thiosemicarbazone, has better antitubercular activity than thiacetazone.

## INTRODUCTION

Mycobacterium tuberculosis, the causative agent for tuberculosis (TB), is responsible for infecting ~10 million people and ~1.5 million deaths in 2020 (1). Despite significant advances in the field, there has been a negligible decline in numbers of new incidence rates and deaths between 2019 and 2020 (1). The situation has worsened as 2.1 million cases of rifampicin-resistant TB (RR-TB) have been reported in 2020, with 6.3% having multidrug-resistant TB (MDR-TB) and 1.2% having extremely drug-resistant TB (XDR-TB) (1). TB treatment is complicated and lengthy, as multiple drugs are used for 6 to 24 months (2). This lengthy and complex treatment regimen is associated with toxicity issues and frequently leads to patient noncompliance. Hence, there is an urgent need to identify shorter treatment options with long-lasting effect that are safe to use and are also effective against DR-TB (1). Recent advances in the research field of TB chemotherapy have led to the approval of bedaquiline (BDQ), delamanid (DLM), and pretomanid (PTM) by the FDA for use in individuals with MDR-TB (3, 4). These molecules inhibit M. tuberculosis growth by inhibiting either ATP synthesis (BDQ) or mycolic acid biosynthesis (DLM or PTM) (5–8). These compounds are being tested in the advanced phase of trials in combination with other new, repurposed, or established drugs to improve their clinical outcomes (1).

TB drugs in the clinical pipeline interfere with well-characterized targets involved in cell wall synthesis, protein synthesis, nucleic acid biosynthesis, oxidative phosphorylation, and cofactor biosynthesis (9–13). Small molecules that target cell wall biosynthesis have been shown to inhibit the growth of both replicating and nonreplicating M. tuberculosis *in vivo* (14, 15). The cell wall inhibitors mostly interfere with the activity of enzymes involved in the biosynthesis of either peptidoglycan or arabinogalactan or mycolic acids (9, 16). Phenotypic screening followed by target validation have identified novel targets impacting major cell wall components, such as DprE1 (target of BTZ043) and MmpL3 (target of SQ109), in addition to earlier established targets, such as InhA, and enzymes involved in peptidoglycan synthesis (17, 18). Moreover, the synergistic effects of carbapenems with rifampicin (RIF); BTZ043 with TMC207; and SQ109 with TMC207, isoniazid (INH), and RIF suggest that cell wall inhibitors contribute to improved efficacy of these drugs in combination (19–23). Overall, these studies show that targeting enzymes involved in mycobacterial cell wall synthesis is one of the well-established strategies to tackle M. tuberculosis infection.

Mycolic acids (MA) are essential components of the M. tuberculosis cell envelope and are important for regulating the acid fastness, permeability, viability, and virulence of M. tuberculosis (24). The MA-containing cell envelope is a low-permeability barrier for various hydrophilic molecules and protects M. tuberculosis against most stresses, including antibiotics. Targeting of this barrier can improve the permeability of antibiotics, resulting in improved TB chemotherapy. INH, thiacetazone (TAC), and ethionamide (ETH) are three TB drugs that are modified by mycobacterial enzymes, and their activated forms interfere with mycolic acid biosynthesis. INH is converted to an active isonicotinoyl radical by mycobacterial catalase peroxidase (KatG, Rv1908c), which binds to NAD^+^ resulting in the formation of an INH-NAD adduct (25, 26). The INH-NAD adduct then binds to NADH-dependent enoyl-acyl carrier protein (ACP) reductase (InhA, Rv1484) of the FAS-II system and inhibits mycolic acid biosynthesis (26–28). TAC is converted to its sulfenic acid form by monooxygenase (EthA, Rv3854c) (29). Subsequently, sulfenic acid binds to FAS-II system’s dehydratase HadAB via a disulphide bound with a cysteine (Cys61) residue of HadA (30, 31). This disulphide bond formation blocks the activity of HadAB, resulting in inhibition of mycolic acid biosynthesis (30, 31). Further, ETH, like TAC is activated by EthA and inhibits InhA, as seen in the case of INH (32–34).

Previously, we had screened a small molecule library from the National Institutes of Health and identified NSC19723 as a compound that possessed activity against Mycobacterium bovis BCG and M. tuberculosis (35). In the present study, NSC19723 was resynthesized, and it displayed MIC value of 0.39 to 0.78 μM against both M. tuberculosis and M. bovis BCG in liquid cultures as previously described (35). Whole-genome sequencing of the resistant strains and computational studies revealed that the mechanism of action of NSC19723 is similar to that reported for TAC. In agreement, mycolic acid biosynthesis was inhibited upon exposure of M. tuberculosis to NSC19723. We show that NSC19723 improved the efficacy of INH in the macrophage and mouse model of infection. Taken together, we have identified a benzaldehyde thiosemicarbazone class of compound that kills M. tuberculosis in a similar way to TAC and has the potential to improve the efficacy of TB drugs.

## RESULTS AND DISCUSSION

### NSC19723 shows specific activity against M. tuberculosis and M. bovis.

Previously, we had screened a small molecule library of 5,000 compounds using M. bovis BCG as a host strain (35). The most active compounds were NSC19723 and NSC18725, which had activity comparable to that of INH in liquid cultures (35). Arora et al. showed that NSC18725 inhibits the growth of intracellular bacteria by inducing autophagy (35). In the present study, we resynthesized NSC19723, a benzaldehyde thiosemicarbazone and evaluated its activity against Mycobacterium smegmatis, ESKAPE (Enterococcus faecium, Staphylococcus aureus, Klebsiella pneumoniae, Acinetobacter baumannii, Pseudomonas aeruginosa, and *Enterobacter* species) pathogens, M. bovis BCG, and M. tuberculosis. As reported earlier, we observed that NSC19723 possessed a MIC value of 0.39 to 0.78 μM against both M. tuberculosis and M. bovis BCG. As shown in Table 1, we observed that NSC19723 and TAC was unable to inhibit the growth of M. smegmatis or ESKAPE pathogens even at 50 μM, the highest concentration tested in the study. The control drugs inhibited the growth of mycobacterial strains and ESKAPE pathogens in the expected range (Table 1). These findings show that NSC19723 specifically inhibits the growth of M. tuberculosis and M. bovis BCG.

**TABLE 1:** Activity of NSC19723 against various microorganisms^*a*^

Strain	MIC value for:			
	NSC19723	Thiacetazone	Tetracycline	Rifampicin
E. coli MSG1655	>50	>50	1.56	6.25
Staphylococcus aureus (ATCC-BAA-976)	>50	>50	0.19	0.024–0.048
Klebsiella pneumoniae (ATCC-33495)	>50	>50	>50	25
Acinetobacter baumannii (ATCC-BAA-2800)	>50	>50	ND^*b*^	25
Pseudomonas aeruginosa (ATCC-2785)	>50	>50	125	25
Enterococcus faecium (ATCC-19434)	>50	>50	0.39	25
M. tuberculosis H37Rv	0.39	0.78–1.56	ND	0.0078
M. bovis BCG Pasteur	0.39–0.78	3.12–6.25	ND	0.0078
M. smegmatis mc^2^ 155	>50	ND	ND	6.25

a The data shown is representative of three experiments performed in duplicates.

b ND, not done.

### NSC19723 displays better profile than TAC in combination with known TB drugs.

**FIG 1:**
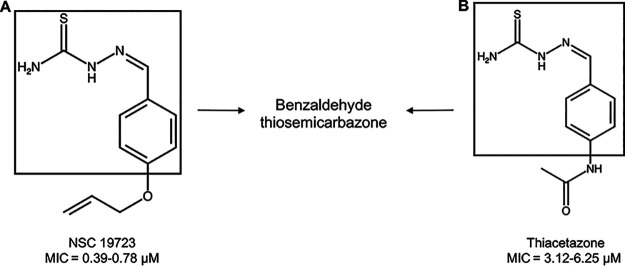
Chemical structure of NSC19723 (A) and TAC (B). MIC values shown are representative of three independent experiments performed in duplicates.

NSC19723 shares structural similarity with TAC, a benzaldehyde thiosemicarbazone (Fig. 1). TAC is a second-line TB drug that had been widely used in Africa and South America to treat TB (36). Previous studies have shown that TAC displays a MIC of 1.56 to 3.125 μM against both M. bovis BCG and M. tuberculosis (37). In subsequent sections, we compared the antitubercular activity of NSC19723 with TAC against M. tuberculosis (38). On the basis of its mechanism of action (MOA), an individual drug used in the combination can either enhance (synergistically) or reduce (antagonistically) the activity of the companion drug or remain indifferent/additive. Within an additive interaction, the effect of two drugs is equal to the sum of the effect of the two drugs taken separately (39).

**FIG 2:**
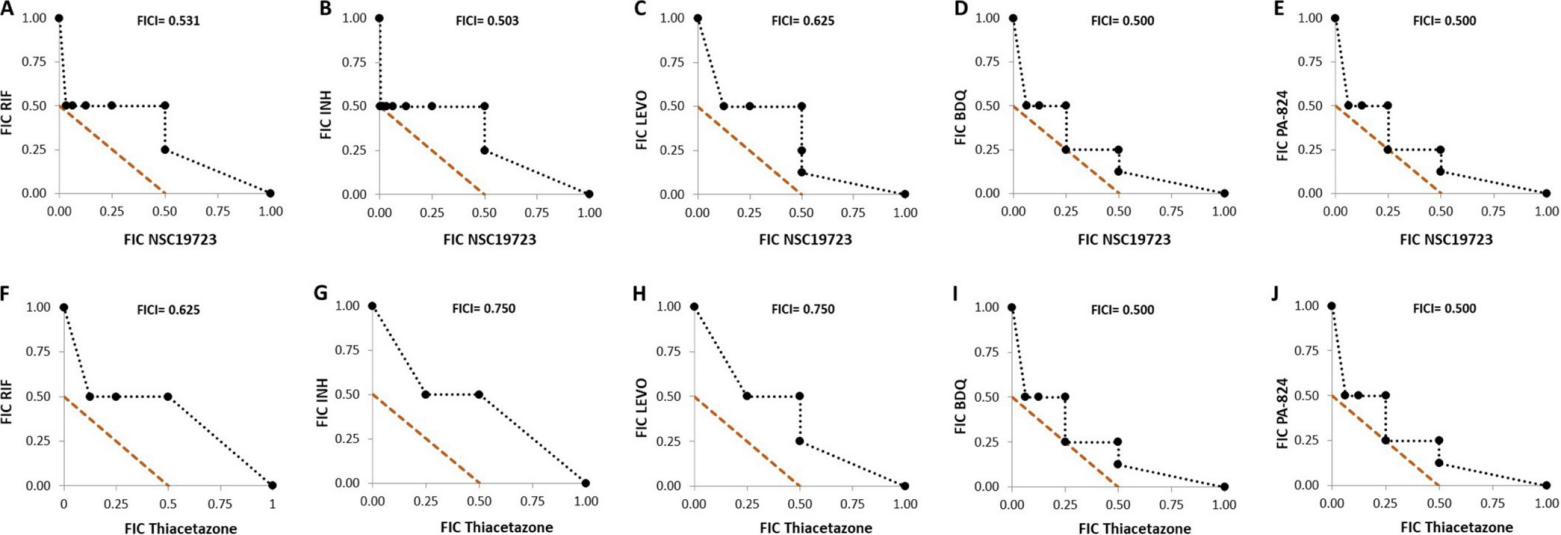
The activity profiles of NSC19723 and TAC in combination with TB drugs against M. tuberculosis. The activity of NSC19723 and TAC was tested in combination with various drugs using checkerboard assays as described in Materials and Methods. FIC values for each drug combination with either NSC19723 (A to E) or TAC (F to J) were determined and shown as isobolograms. The data shown for checkerboard assays are representative of two independent experiments performed in duplicate.

We next determined the antimycobacterial activity of NSC19723 on the growth of M. tuberculosis in combination with known TB drugs. In our checkerboard assays, we observed that NSC19723 and TAC synergise with BDQ or PA-824, with fractional inhibitory concentration index (FICI) values of 0.5 for each drug (Fig. 2D to J). We observed indifferent interaction when NSC19723/TAC was combined with RIF or INH or Levofloxacin (Levo), with the FICI values of 0.531/0.625 with RIF, 0.503/0.75 with INH, and 0.625/0.75 with Levo, respectively (Fig. 2A to H). Interestingly, in combination with INH, NSC19723 inhibited the growth of M. tuberculosis at a concentration of 0.0015 μM. However, in combination with BDQ/PA-824, NSC19723 was able to inhibit the growth of M. tuberculosis at only 0.097 μM (see Table S1 in the supplemental material). In comparison, in combination with INH or BDQ or PA-824, 0.391 μM TAC was required to inhibit the growth of M. tuberculosis (see Table S2 in the supplemental material). These findings also reveal that combining NSC19723 with TB drugs improved the MIC of RIF 2.0-fold and of INH/Levo/BDQ/PA-824 each by 4.0-fold against M. tuberculosis (Table S1).

### NSC19723 improves the killing activity of known TB drugs in liquid cultures and macrophages.

**FIG 3:**
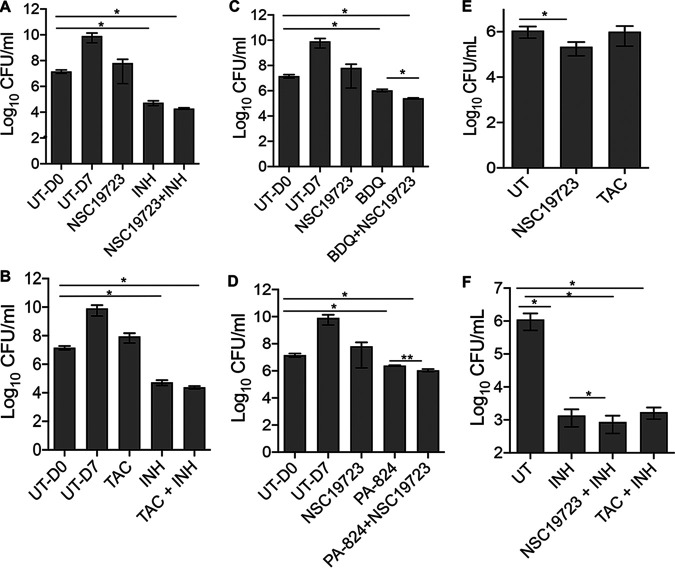
Antimycobacterial activity of NSC19723 and TAC either alone or in combination with known TB drugs in liquid cultures and macrophages. (A to D) Early logarithmic phase (OD_600_, ~0.2) cultures of M. tuberculosis were exposed to 10× MIC of NSC19723/TAC alone and in combination with various drugs for 7 days. The data shown in these panels are means ± standard deviation (SD) of log_10_ CFU obtained from three independent experiments. The same UT-, NSC19723-, or INH-treated panels have been used as controls. (E and F) For intracellular killing experiments, THP-1 cells were infected with M. tuberculosis at an MOI of 1:10. The cells were overlaid with various drugs either alone or in combination for 4 days. The data shown in these panels are means ± SD log_10_ CFU in different conditions obtained from two independent experiments performed in either duplicates or triplicates. Using the paired *t* test (two-tailed), statistically significant differences were found between the indicated groups; *, *P* < 0.05; **, *P* < 0.01. The same “UT” panel has been used as control. The limit of detection for CFU assays in panels A to F is 40 CFU/mL.

Early bactericidal activity (EBA) has an important role in predicting the bacteriostatic/bactericidal nature of a combination therapy (40, 41). The ideal antitubercular agent should exhibit an early bactericidal activity to minimize the risk of resistance development. We next determined the mode of mycobacterial killing by NSC19723 in liquid cultures. The activity of NSC19723 was evaluated alone and in combination with INH, BDQ, or PA-824 in 7H9 medium against M. tuberculosis H37Rv. We observed that the bacterial counts after exposure to either NSC19723 or TAC at 10× MIC for 7 days were comparable to those of untreated cultures (day 0 readings) (Fig. 3A and B). The growth of M. tuberculosis was significantly reduced by 250.0-fold when exposed to 10× MIC of INH (*, *P* < 0.05). As shown in Fig. 3A and B, the activity of INH further increased by ~2.0- to 3.0-fold in combination with either NSC19723 or TAC. We also compared the activity of BDQ/PA-824 either alone or in combination with NSC19723 against M. tuberculosis in liquid cultures. We observed that MIC values of BDQ and PA-824 against M. tuberculosis were 0.4 μM. As shown in Fig. 3C and D, we observed that exposure of early-log-phase cultures to either BDQ or PA-824 reduced the growth 13.0-fold and 6.0-fold, respectively (*, *P* < 0.05). We also observed that in combination with NSC19723, the *in vitro* activity of BDQ and PA-824 was increased by 4.0- and 2.0-fold, respectively (Fig. 3C and D; *, *P* < 0.05; **, *P* < 0.01).

TB infection starts when M. tuberculosis reaches the alveolar air sacs of the lungs and macrophages clear the intracellular pathogen through the process of phagocytosis. We also compared the ability of NSC19723 either alone or in combination with INH to inhibit the growth of intracellular bacteria. The concentrations of drug that reduced viability of macrophages by 50% compared to untreated control macrophages (TC_50_) of NSC19723 and INH against THP-1 macrophages were >50 μM in our cell viability experiments. In agreement with *in vitro* activity data, exposure to NSC19723 slightly inhibited the growth of intracellular M. tuberculosis by 5.0-fold compared to that of dimethyl sulfoxide (DMSO)-treated cells (Fig. 3E; *, *P* < 0.05). Surprisingly, we did not observe any growth inhibition of intracellular M. tuberculosis after exposure to TAC. As expected, INH treatment inhibited the growth of intracellular M. tuberculosis by ~820.0-fold (Fig. 3E; *, *P* < 0.05). This killing activity of INH increased to ~1,300.0-fold in combination with NSC19723 (Fig. 3F; *, *P* < 0.05). We also observed that INH-mediated killing of intracellular bacteria did not improve in combination with TAC (Fig. 3F). Our findings suggests that NSC19723 has the potential to improve the efficacy of BDQ and PA-824 against M. tuberculosis *in vitro* and of INH in macrophages.

### NSC19723 and thiacetazone possess similar mechanism of action against M. tuberculosis.

Further, we performed experiments to gain further insights into the mechanism of action of NSC19723. We observed that MIC values of NSC19723 against M. bovis BCG and M. tuberculosis were comparable to each other (Table 1). The genome of M. bovis BCG is ~99% similar to the M. tuberculosis genome. Since, M. bovis BCG is a biosafety level II (BSL-II) pathogen and easier to handle, we next attempted to generate spontaneous resistant strains against NSC19723 in M. bovis BCG. We were able to raise M. bovis BCG Pasteur strains that were resistant to NSC19723. We observed that the *in vitro* frequency of resistance against NSC19723 to be 2 × 10^−7^. This mutation frequency was similar to the frequency of resistance against TAC and INH (42, 43). As shown in Table 2, MIC of NSC19723 against the resistant strain was increased >64.0-fold in comparison to the MIC values obtained for the parental strain. As expected, the resistant strains were as susceptible as the parental strain to standard anti-TB drugs, such as RIF, INH, and Levo (Table 2). In order to identify the cellular target for NSC19723, genomic DNA was isolated from both parental and resistant strains. The isolated genomic DNA was subjected to whole-genome sequencing for identification of single nucleotide polymorphisms (SNPs) linked to a resistance phenotype. The analysis of the sequencing results revealed that the resistant mutant strain harbours a missense mutation (367^A→G^) in the *hadC* gene. The amino acid was changed of Thr^123^ to Ala^123^ as a result of this missense mutation.

**TABLE 2:** Cross-resistance of profile of NSC19723-resistant mutants

Treatment	MIC (μM)^*a*^			
	M. bovis BCG Pasteur-WT	M. bovis BCG Pasteur-NSC19723^r^ HadC^T123A^	M. bovis BCG-pVV16	M. bovis BCG-pVV16-hadABC
NSC19723	0.39–0.78	>50	0.78	25–50
Thiacetazone	3.12–6.25	>50	3.12–6.25	>50
Isoniazid	0.39	0.39	0.39	0.39
Rifampicin	0.0039–0.0078	0.0078	ND	ND
Levofloxacin	0.39	0.39	ND	ND
Ethionamide	12.5–25	12.5	ND	ND

a The data shown is representative of three experiments performed in duplicates. WT, wild type; NSC19723^r^, NSC19723 resistant; ND, not done.

**FIG 4:**
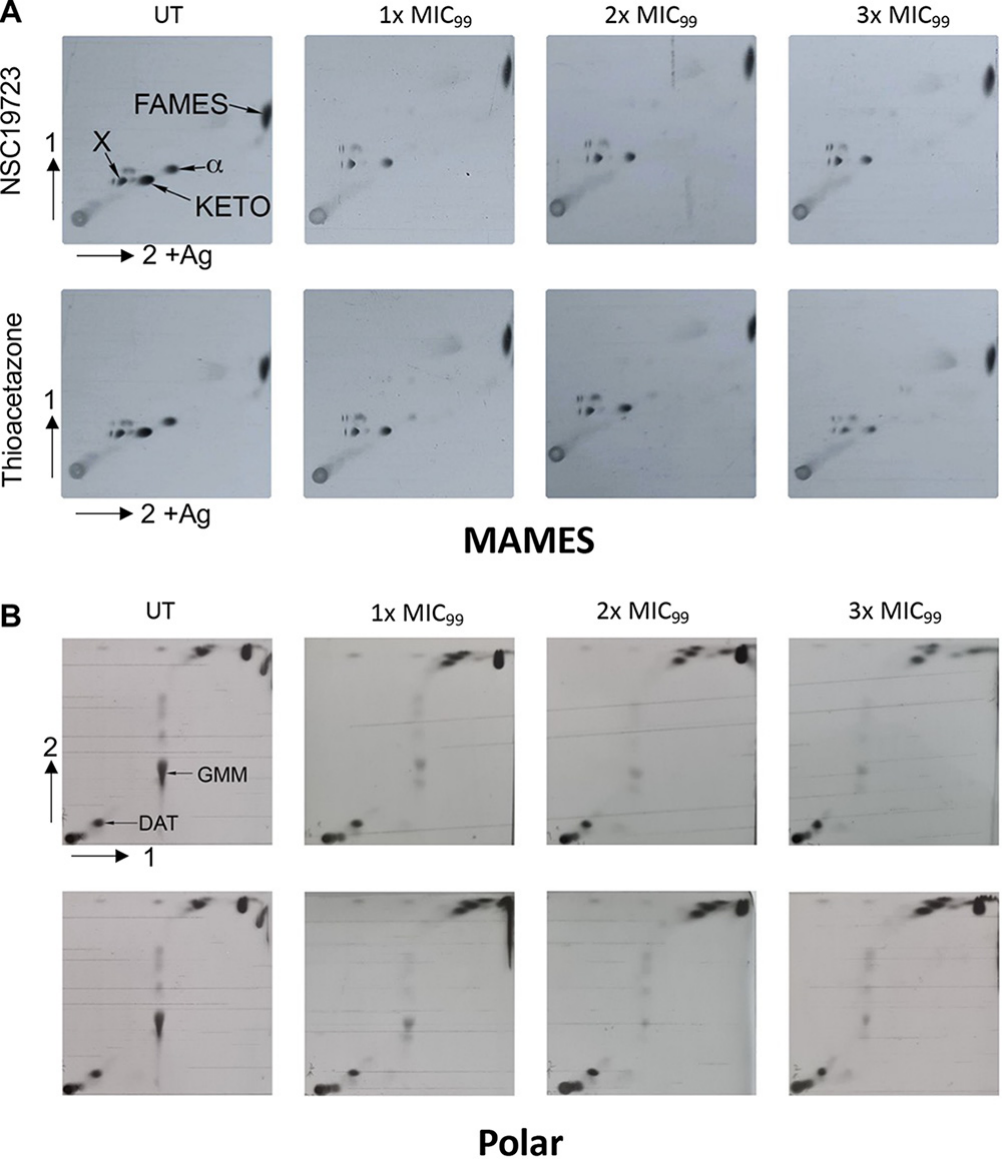
[14C]-Acetate labeling and dose response of NSC19723 and TAC against M. bovis BCG cultures. (A) Total FAMEs and MAMEs were extracted and equal counts (50,000 cpm) analyzed by 2D-Ag^+^ TLC and exposed to a Kodak BioMax MR film for 5 days, with comparison to untreated cultures (UT). Two-dimensional (2D) Ag^+^ silica TLC was performed using the solvent system to resolve FAMEs/MAMEs and X as follows: first direction, two developments of hexane/ethyl acetate (95:5, vol/vol); second direction, three developments of petroleum ether/diethyl ether (85:15 vol/vol). (B) Lipid profiles of M. bovis BCG (UT) and M. bovis BCG treated with NSC19723 and TAC as determined by 2D-TLC. Polar lipids were isolated, and an equal aliquot (50,000 cpm) for each sample was subjected to 2D-TLC in a solvent system that resolves GMM as follows: first direction, chloroform/methanol/water (100:14:0.8, vol/vol/vol); second direction, chloroform/methanol/acetone/water (50:60:2.5:3, vol/vol/vol/vol) and exposed to a Kodak X-Omat film for 5 days. FAMEs, fatty acid methyl esters; MAMES, α, α-mycolic acid methyl esters; KETO, ketomycolic acid methyl esters; X, unsaturated ketomycolic acid precursor; GMM, glucose monomycolate; DAT, diacyl trehalose. The data shown in this panel is representative of two experiments. Same “UT” panel has been used as control for both NSC19723- and thiacetazone-treated panels.

HadC is a member of the essential Rv0635-Rv0636-Rv0637 locus that encodes for hot dog folded proteins, HadA, HadB, and HadC (44). These proteins function via forming heterodimers HadAB and HadBC dehydratases and are a component of the M. tuberculosis fatty acid synthase type II (FAS-II) system (24). Earlier studies involving TAC-resistant strains have also reported mutations in the HadABC complex. These mutations include HadA^C61S^, HadC^T123A^, HadC^V85I^, HadC^K157R^, or HadC^A151V^ (30, 31, 42, 45). HadAB dehydrates short meromycolyl precursors during the early stages of their elongation by FAS-II as demonstrated by phenotypic analysis of an HadC-deficient strain. HadBC, conversely, dehydrates long-chain fatty acyl substrates. In agreement, NSC19723-resistant strains showed >16.0-fold resistance to TAC (Table 2). Previous studies have demonstrated that TAC and ETH are prodrugs, which are activated by flavin-dependent monooxygenase EthA to exert their antimycobacterial activity (32). Since the mutations in NSC19723-resistant mutants mapped to HadC, these strains were as sensitive as the parental strain toward ETH, and a MIC value of 12.5 to 25 μM was observed (Table 2). Further, MIC determination assays for parental and HadABC overexpression strains were also performed. As shown in Table 2, we observed that the overexpression strain was also resistant to NSC19723 and TAC. We observed MIC values of 25 to 50 and >50 μM for NSC19723 and TAC, respectively, against the M. bovis BCG HadABC overexpression strain (Table 2). Next, we compared the lipid profiles of M. bovis BCG upon exposure to either NSC19723 or TAC at 1×, 2×, or 3× MIC. NSC19723 and TAC both strongly inhibited mycolic acid biosynthesis in a dose-dependent manner, resulting in inhibition of all classes of mycolic acid methyl esters (MAMES) (Fig. 4A). Similarly, polar lipid analysis revealed that exposure to both drugs inhibits the synthesis of the mycolate-containing glycolipid glucose monomycolate (GMM) in a dose-dependent manner (Fig. 4B). The levels of fatty acid methyl esters (FAMES) and diacyl trehalose (DAT) in untreated and treated samples were comparable (Fig. 4A and B). These results suggest that NSC19723, like TAC, has an effect on the dehydration step in the mycolic acid biosynthesis.

**FIG 5:**
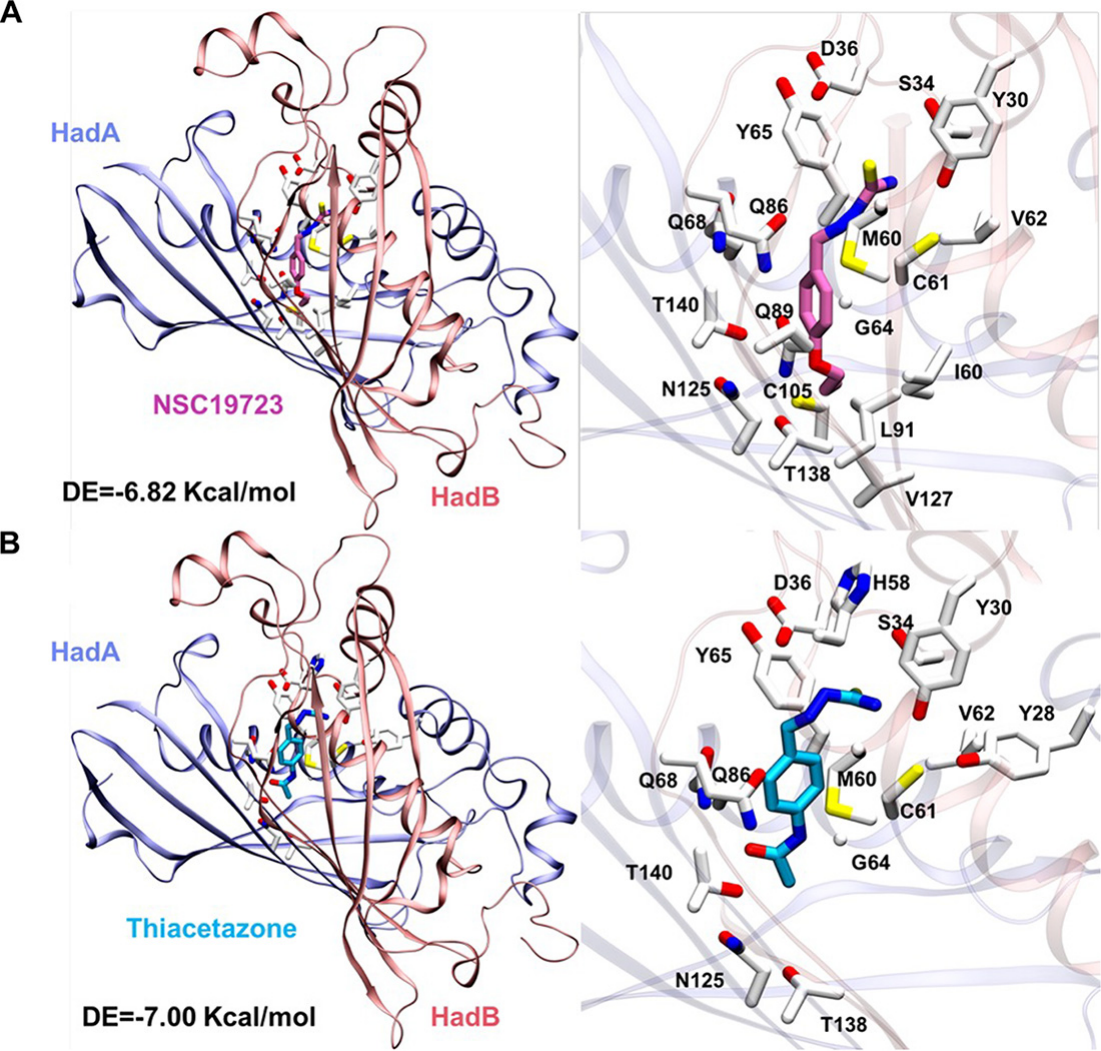
Interaction pattern of the compounds identified from molecular docking. The most likely binding pose of NSC19723 (A) and TAC (B) with HadAB identified from the first cluster. The residue-wise interactions of HadAB proteins with NSC19723 and TAC. All amino acids are rendered in licorice and shown in atom-wise mode (C, white; O, red; N, blue; S, yellow). The compounds are also shown in atom-wise (C, pink/cyan), respectively, while the rest of the atoms remain the same. The residues within 3.5 Å from the compounds binding pose are shown for interactions. Proteins (chain A, ice-blue; chain B, fluorescent red) are color transparent, and chemicals are labeled with their respective colors.

Further, molecular docking studies were performed to better understand the binding pattern and interactions of NSC19723 and TAC with the HadAB protein. Using AutoDock, the top three clusters of the ligands that are substantially populated with the number of conformations at the active site were selected for further analysis. The first cluster in the docking simulation exhibited the least energy and had the highest population of conformations for all three ligands (Table 3). Based on the binding energies and number of conformations in clusters, the first cluster of compounds was analyzed. As shown in Table 3, the binding energy for NSC19723 and TAC with first cluster ranked conformers was −7.02 and −6.54, respectively. The number of conformations obtained for NSC19723 and TAC with HadAB in cluster 1 were 57 and 34, respectively (Table 3). We observed that both NSC19723 and TAC fitted well in the binding groove of HadAB, which is majorly covered by polar and hydrophobic residues (Fig. 5A and B and Table 4). The residues such as Cys61, Gly64, Tyr65, Gln68, Gln86, Asn125, and Thr140 of HadA and Asp36 and Met60 of HadB were implicated in binding for both NSC19723 and TAC (Fig. 5A and B; Table 4). These findings are in concordance with previously published work (30). However, some additional residues from both subunits were also identified for the binding of NSC19723 and TAC. HadA’s Ile60, Val62, Leu91, Val127, and Thr138 as well as HadB’s Tyr30 and Ser34 were found to interact with NSC19723 (Fig. 5A). Similarly, Tyr28 and Thr138 of HadA and Tyr30, Ser34, and His58 of HadB proteins were found to bind to TAC (Fig. 5B). These additional interacting residues are most likely responsible for the distinct binding energies obtained for NSC19723 and TAC (Fig. 5A and B; Table 3). Taken together, we conclude that HadAB protein interacts with NSC19723 and TAC in a similar manner.

**TABLE 3:** The docking clusters along with conformers and docking energies^*a*^

Compound	Cluster rank	No. of conformers	Docking energy (kcal/mol)
NSC19723	**1**	**57**	**−7.02**
	2	54	**−**6.55
	3	42	**−**6.54
Thiacetazone	**1**	**34**	**−6.54**
	2	25	**−**6.28
	3	22	**−**6.08

a The first cluster (bold) is chosen for further analysis.

**TABLE 4:** Interacting residues of HadAB with NSC19723 and thiacetazone

Compound	Chain	Binding site residues type			
		Acidic	Basic	Polar	Hydrophobic
NSC19723	HadA			G64, Y65, Q68, Q86, Q89, N125, T138, T140	I60, C61, V62, L91, C105, V127,
	HadB	D36		Y30, S34	M60
Thiacetazone	HadA			Y28, G64, Y65, Q68, Q86, N125, T138, T140	C61, V62,
	HadB	D36	H58	Y30, S34	M60

### NSC19723 improves the efficacy of frontline TB drug in mouse model of TB infection.

**FIG 6:**
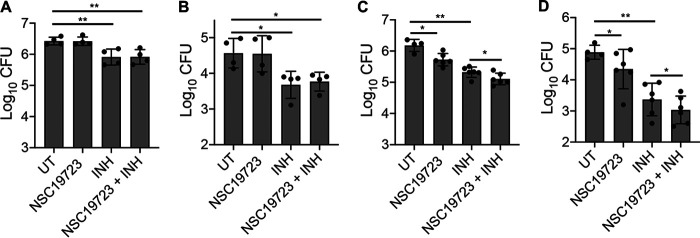
Antitubercular activity of NSC19723 either alone or in combination with INH in mouse model of infection. Male or Female BALB/c mice were infected via aerosol route, and drugs were administered after 4 weeks of infection. The lung and splenic bacillary loads were determined at 2 weeks (A and B) and 4 weeks (C and D) posttreatment. The data shown in these panels are mean ± SD of log_10_ CFU obtained from either 4 or 6 animals. Using the paired *t* test (two-tailed), significant differences were obtained between the indicated groups; *, *P* < 0.05 and **, *P* < 0.01. The limit of detection for these assays is 80 CFU/mL.

Previously, Jagannath et al. demonstrated that administration of 60 mg/kg TAC treatment for 4 weeks resulted in an ~10.0-fold reduction in both lung and splenic bacillary load in C57B/6 mice infected with M. tuberculosis Erdman (46). In this study, the mice were infected via intravenous route, and administration of TAC was initiated 24 h postinfection. However, in another study, Fattorini et al. showed that administration of 60 mg/kg TAC for 4 weeks resulted in 1.9-fold and 1.6-fold reduction in lung and spleen bacillary load, respectively, in M. tuberculosis M22-infected BALB/c mice (47). We also evaluated the efficacy of NSC19723 in aerosol-infected mice, but the treatment was initiated at 28 days posttreatment. Before performing the efficacy experiment, we performed a dose tolerance assay for NSC19723 to determine an efficacy dose. For this, mice were administered with vehicle (PEG 400) and 100, 75, or 50 mg/kg of NSC19723 for a week, and various health parameters were monitored. The mice gavaged with 100 mg/kg of NSC19723 showed hyperactivity; however, mice gavaged with 75- or 50-mg/kg doses showed no behavioral abnormalities. Therefore, we selected a 75-mg/kg dose of NSC19723 for mice efficacy experiments. We evaluated the efficacy of NSC19723 either alone or in combination with INH following administration of drugs for either 2 weeks or 4 weeks. The aerosol infection led to the implantation of ~100 M. tuberculosis CFU at day 1 postinfection. At 2 weeks posttreatment, exposure to INH reduced the lungs bacillary loads by 3.0-fold (Fig. 6A; **, *P* < 0.01). In agreement, treatment of animals with INH for 2 weeks reduced the splenic bacterial loads by 7.5-fold (Fig. 6B; *, *P* < 0.05). We did not observe any inhibition of M. tuberculosis growth in both lungs and spleen tissues upon exposure to NSC19723 after 2 weeks of treatment. Further, the activity of INH did not increase upon coadministration with NSC19723 after 2 weeks of treatment (Fig. 6A and B). As shown in Fig. 6C, in comparison to untreated mice, treatment with either INH or NSC19723 for 4 weeks reduced the lung bacillary loads by ~7.0- and 3.0-fold, respectively (*, *P* < 0.05; **, *P* < 0.001). Also, coadministration of NSC19723 along with INH further improved its activity in lung tissues by ~2.0-fold (Fig. 6C; *, *P* < 0.05). In agreement, the splenic bacillary loads in INH-treated mice were reduced by 32.0-fold compared to those of untreated mice (Fig. 6D; **, *P* < 0.01). In a combination of INH with NSC19723, ~70.0-fold reduction in splenic bacillary load was observed in spleens at 4 weeks posttreatment. This ~2.0-fold reduction in the combination group relative to the INH-treated group was observed to be statistically significant (Fig. 6D; *, *P* < 0.05). We observed that daily administration of NSC19723 also reduced the bacterial growth by 3.5-fold in spleens of infected animals (Fig. 6D; *, *P* < 0.05). Taken together, these findings imply that NSC19723 treatment marginally inhibited M. tuberculosis growth in animal tissues but improved the activity of INH in chronic model of infection.

### Conclusion.

**FIG 7:**
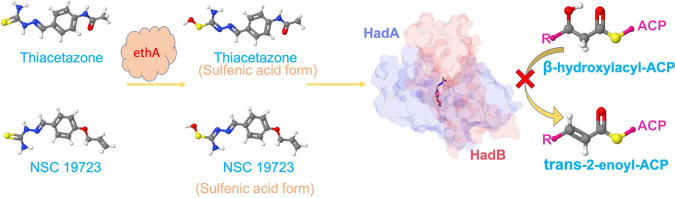
Mechanism of action of NSC19723. NSC19723, like TAC, is a benzaldehyde thiosemicarbazone class prodrug. The activation of this drug requires metabolic oxidation of their thiocarbonyl moiety by the flavin-dependent monooxygenase, EthA. NSC19723’s activated sulfenic acid form binds to HadAB (FAS-II-hydroxyacyl ACP dehydratase) and inhibits the formation of *trans*-2-enoyl-ACP from β-hydroxyacyl ACP. This subsequently leads to inhibition of mycolic acids upon exposure of M. tuberculosis to NSC19723.

M. tuberculosis inhibitors, which compromise cell wall architecture, not only kill but also improve the intrabacterial concentrations of other TB drugs. TAC and INH combination has been successful for treatment of individuals with MDR-TB (36). However, it was subsequently removed from the anti-TB chemotherapeutic regimen due to the associated secondary toxic effects, especially in HIV-positive patients (48). Our findings show that NSC19723, a molecule belonging to the chemical class of TAC, has better antimycobacterial activity than TAC in various models of anti-TB activity. NSC19723 is a benzaldehyde thiosemicarbazone class of prodrug. The flavin-dependent monooxygenase, EthA, might be required for the activation of NSC19723. The activated sulfenic acid form of NSC19723 interacts with HadAB (FAS-II-hydroxy acetyl ACP dehydratase) and prevents conversion of hydroxyacyl ACP to trans-2-enoyl-ACP (Fig. 7). Therefore, similar to TAC, exposure to NSC19723 also results in the suppression of mycolic acid biosynthesis in M. tuberculosis. Our work also shows that NSC19723 has the potential in terms of reducing the dosages of INH and BDQ/PA-824, two recently approved FDA drugs. We did not evaluate this molecule for possible toxic effects, as it was beyond the scope of this investigation. Future work would involve studying the efficacy of this molecule with BDQ or PA-824, pharmacokinetic/pharmacodynamic profile of this molecule, and drug-drug interactions with known TB drugs.

## MATERIALS AND METHODS

### Media and reagents.

Cell culture reagents were procured from Gibco Laboratories (Grand Island, NY, USA). TX-100, Tween 80, and phorbol 12-myristate 13-acetate (PMA) were purchased from Sigma (St. Louis, MO, USA). Middlebrook growth media 7H9 broth, 7H10 and 7H11 agar, Mueller-Hinton broth, brain heart infusion broth, tryptic soy broth, and Luria-Bertani (LB) broth were procured from Becton, Dickinson (MD, USA).

### Bacterial strains and culture conditions.

Mycobacterial strains (M. tuberculosis H37Rv ATCC-27294, M. bovis BCG Pasteur, and M. smegmatis mc^2^ 155) were grown in Middlebrook 7H9 broth (MB broth) supplemented with Tween 80 (0.05%), glycerol (0.2%), and albumin-dextrose-saline (ADS) (1.0×) with shaking at 200 rpm and 37°C. ESKAPE pathogens were cultured in Mueller-Hinton broth (Pseudomonas aeruginosa ATCC-2785, Klebsiella pneumoniae ATCC-33495, and Staphylococcus aureus ATCC-BAA-976), brain heart infusion broth (Enterococcus faecium ATCC-19434), tryptic soy broth (Acinetobacter baumannii ATCC-BAA-2800), and Luria-Bertani broth (Escherichia coli MSG1655) with shaking at 200 rpm and 37°C. For CFU enumeration, bacterial cultures were serially diluted in phosphate-buffered saline (PBS) and plated on Middlebrook 7H11 agar (MB agar) supplemented with glycerol (0.5%) and oleic-albumin-dextrose-saline (OADS) (1×) followed by incubation at 37°C for 3 to 4 weeks.

### Chemical synthesis of NSC19723.

The details of the synthesis of NSC19723 are described in the Supplementary Methods found in the supplemental material. The chemicals for the synthesis of NSC19723 were purchased from Sigma-Aldrich. The formation of products in various chemical reactions was monitored by thin-layer chromatography (TLC). The final compound was resuspended in DMSO-d^6^, and ^1^H-nuclear magnetic resonance (NMR) and ^13^C-NMR data were collected to determine purity.

### MIC determination assay.

MIC values of compounds against all strains were determined using the broth microdilution method as previously described (49). Briefly, 2-fold serial dilutions of drugs were prepared in 96-well clear “U” bottom plates followed by the addition of an equal volume of 1:1,000 times diluted early-logarithmic bacterial culture (optical density at 600 nm [OD_600_], ~0.2). The plates were incubated at 37°C for either 14 days (for M. tuberculosis), 2 days (for M. smegmatis), or 1 day (for ESKAPE pathogens). The MIC value for a particular drug was reported as a lowest concentration at which no visible growth was seen in the form of a pellet.

### Synergy assays.

Drug combination experiments were performed by two-drug checkerboard assays in 96-well clear U bottom plates. The compounds (NSC19723 and TAC) were diluted horizontally 2.0-fold, and known TB drugs were diluted vertically 2.0-fold to prepare various drug combinations. Further, early-logarithmic culture of an OD_600_ of ~0.2 of M. tuberculosis was diluted 1:1,000 times and added to the plates followed by incubation at 37°C for 14 days. Synergistic, indifferent, or antagonistic interactions for various drug combinations were determined by calculating fractional inhibitory concentration (FIC) and fractional inhibitory concentration index (FICI) using the below mentioned formula as described earlier (50).

∑FICI=FIC(A)+FIC(B)

where

$$\text{FIC}\left(\text{A}\right)=\frac{\text{MIC}\left(\text{A}\right)\text{ in combination}}{\text{MIC}\left(\text{A}\right)\text{ alone}}$$

$$\text{FIC}\left(\text{B}\right)=\frac{\text{MIC}\left(\text{B}\right)\text{ in combination}}{\text{MIC}\left(\text{B}\right)\text{ alone}}$$

### Cell viability assay.

Roswell Park Memorial Institute (RPMI) medium supplemented with heat-inactivated fetal bovine serum (HI-FBS) (10%) was used to culture the THP-1 cell line at 37°C and 5% CO_2_. PMA at a concentration of 20 ng/mL was added to differentiate the monocytes into macrophages. For cell viability assay, THP-1 cells (2 × 10^4^/well) were seeded in 96-well flat-bottom plates in the presence of PMA. After 48 h of incubation, the monolayer was washed twice with 1× PBS and overlaid in RPMI medium (10% HI-FBS) for 24 h. The dilutions of various drugs were prepared in RPMI medium (10% HI-FBS) and added in triplicates. After 96 h of incubation, cell proliferation reagent (WST-1) was added to each well. After 30 min of incubation, the absorbance at 450 nm and 630 nm was measured. The percentage of cell viability in the presence of drugs (TC_50_) was calculated as per manufacturer’s recommendations. TC_50_ was defined as the concentration of drug that reduced viability of macrophages by 50% compared to untreated control macrophages.

### Antimycobacterial killing experiments in liquid cultures and macrophages.

For *in vitro* killing assay, early-log-phase (~0.2 OD_600_) cultures of M. tuberculosis were exposed to 10× MIC of various drugs for 7 days. For intracellular killing experiments, THP-1 cells (2 × 10^5^/well/mL) were seeded in a 48-well cell culture plate in the presence of PMA. Following 48 h of incubation, the monolayer was washed twice with 1× PBS and overlaid in RPMI medium (10% HI-FBS) for 24 h. Subsequently, macrophages were infected with single-cell M. tuberculosis suspension with a multiplicity of infection (MOI) of 1:10 (2 × 10^6^ bacilli/well). After 4 h of infection, extracellular M. tuberculosis was removed by overlaying macrophages with RPMI containing 200 μg/mL of amikacin for 2 h. Further, macrophages were washed twice with 1× PBS and overlaid with RPMI containing compounds/drugs. The samples were collected at day 0, day 2, and day 4 by lysing macrophages in 1 mL 1× phosphate-buffered saline with triton X-100 (PBST) (0.1% Triton X-100), and bacterial enumeration was performed as mentioned above.

### Generation of resistant mutant strains and whole-genome sequencing.

NSC19723 spontaneous resistant mutants were generated as described earlier by Kidwai et al. with slight modifications (51). In brief, mid-log-phase culture of M. bovis BCG Pasteur (OD_600_, ~1.0) was plated on MB agar plates containing the NSC19723 at 10× MIC. The colonies that appeared on these drug-containing plates were subcultured, and MIC values were determined as described above. The colonies that displayed at least a 4.0-fold increase in MIC values were subjected to whole-genome sequencing. For identification of genes linked with resistance, genomic DNA was isolated from various strains and shipped to Clevergene, Biocorp Pvt Ltd, India for next-generation sequencing and subsequent analysis. Briefly, whole-genome sequencing libraries were prepared using NEBNext Ultra II FS DNA library prep kit for Illumina. The samples were purified using 0.8× AMPure XP beads, and the DNA was amplified using NEBNext Ultra II Q5 master mix for 6 cycles of PCR. The NEBNext Multiplex Oligos for Illumina kit was used for multiplexing. The Agilent HS D1000 ScreenTape System was used to assess the library’s quality. The Agilent 4150 TapeStation system was used to run the samples, and the Agilent TapeStation analysis software was used to analyze them. Quality trimmed reads were aligned to the reference genome of M. bovis BCG Pasteur using Bowtie 2 with default parameters. Freebayes v1.3.1 was used to call variants, and these were annotated using snpEff.

### Generation of HadABC overexpression M. bovis BCG.

For overexpression studies, *hadABC* was PCR amplified using gene-specific primers (HadA-F, GCATATGGTGGCGTTGAGCGCAGACATCGTTGGG; HadC-R, GAAGCTTTTACGCGGTCCTGATGACCTGCCCG) and cloned into the mycobacterial expression vector, pVV16. The construction of the recombinant plasmid was verified by sequencing. The overexpression strains were prepared by electroporation of pVV16-*hadABC* in M. bovis BCG Pasteur followed by the selection of transformants on 7H11 medium supplemented with kanamycin (25 μg/mL) at 37°C for 3 to 4 weeks.

### Radioactive labeling analysis of lipids, fatty acid methyl esters, and mycolic acid methyl esters.

M. bovis BCG were grown to an OD_600_ of ~0.4 and then treated for 24 h at 37°C with compounds/drugs (TAC and NSC19723) at multiple concentrations (1×, 2×, and 3× MIC). Cultures were then radiolabeled using [1,2-^14^C] acetic acid (10 μCi, 50 to 62 mCi mmol^−1^; Perkin Elmer) and incubated at 37°C for another 24 h. The culture was subsequently divided into two equal 5-mL aliquots. In order to isolate nonpolar and polar lipids, one aliquot was treated to organic extraction as reported earlier (52, 53). The samples (equal counts, typically 50,000 cpm) were applied to silica gel plates, and two-dimensional TLC was performed. The solvent system to resolve GMM in the first direction was chloroform/methanol/water (100:14:0.8, vol/vol/vol), and chloroform/methanol/acetone/water (50:60:2.5:3, vol/vol/vol/vol) was used for second direction. Further, TLC plates were exposed by autoradiography to Kodak BioMax MR film for 5 days. Labeled GMM was further quantified by imaging and compared with known standards.

In order to isolate the combined fatty acid methyl esters (FAMEs) and mycolic acid methyl esters (MAMEs) (alpha and keto), the second aliquot was treated with alkaline hydrolysis, followed by methylation as previously described (52, 53). The lipids were resuspended in 200 μL of chloroform. An aliquot of 5-μL lipid sample was removed, dried in scintillation vials, and then resuspended in 10 mL of scintillation fluid (Ecoscint A; National Diagnostics) and counted on a Tri-Carb 2700TR liquid scintillation analyser. In order to resolve FAMEs/MAMEs and X, equal counts of samples (~50,000 cpm) were loaded to silica gel plates, and two-dimensional Ag^+^ silica TLC was performed. For the first direction, two developments of hexane/ethyl acetate (95:5, vol/vol/vol) were performed, and for the second direction, three developments of petroleum ether/diethyl ether (85:15 vol/vol) were performed. The TLC plate was processed as described above, and the results were compared to recognized standards.

### Molecular modeling and docking studies.

The crystal structure of the HadAB protein (PDB ID number 4RLJ) was obtained from the PDB database (https://www.rcsb.org/). Both of the chains HadA and HadB were selected for molecular docking experiments. The structures of the ligands, NSC19723, and TAC were optimized using the Biovia Discovery Studio 2020 for docking studies as previously described. The AutoDock tool 1.5.6 suite was used to simulate the docking of ligands with the HadAB protein (54, 55). Prior to docking studies, HadAB was prepared by adding polar hydrogen atoms, kollaman charges, and AD4-type atoms. The ligands were prepared by first identifying the root and then by setting up the number of torsions and the partial charge. The grid was established along *x*, *y*, and *z* coordinates with a spacing of 0.547 Å to completely cover the active site of HadAB protein (30). The Lamarckian genetic algorithm (LGA) was used to simulate docking of small molecules with all default parameters except GA runs, which were set to 200 and energy assessment steps, which were set to 2.5 × 10^7^.

### Animal efficacy experiment.

The animal experimental procedures were performed in an animal biosafety level III facility of the Translational Health Science and Technology Institute and approved by the Institutional Animal Ethics Committee. For drug efficacy experiments, mice were infected via aerosol route with approximately 100 CFU of M. tuberculosis H37Rv. After 4 weeks postinfection, four (2 male and 2 female) or six mice per group (3 male and 3 female) were treated with either 75 mg/kg NSC19723, 10 mg/kg isoniazid, or 75 mg/kg NSC19723 plus 10 mg/kg isoniazid for either 2 weeks or 4 weeks. The drugs were administered daily via oral route in PEG 400 formulations for 6 days a week. Lungs and spleens were homogenized in saline, and 10-fold serial dilutions was plated on MB 7H11 agar plates at 37°C for 3 to 4 weeks for CFU enumeration.

### Statistical analysis.

Prism 8 (version 8.4.3) was used for preparation of graphs and statistical analysis. Statistical significance has been shown in figures and figure legends. A *P* value of <0.05 between the indicated group using paired *t* test (two-tailed) was considered to be significant.
