# Supplementary material for: NSC19723, a Thiacetazone-Like Benzaldehyde Thiosemicarbazone Improves the Efficacy of TB Drugs In Vitro and In Vivo
Source: Microbiol Spectr. 2022 Oct 31;10(6):e02592-22. doi: 10.1128/spectrum.02592-22 (PMC9769743; doi:10.1128/spectrum.02592-22)
Supplement: Supplemental file 1 — Supplemental material. Download spectrum.02592-22-s0001.pdf, PDF file, 0.5 MB [file spectrum.02592-22-s0001.pdf]

Supplementary Methods

Scheme and procedure for synthesis of NSC 19723 i.e. *(E)-2-(4-(allyloxy)benzylidene) hydrazine-1-carbothioamide*:

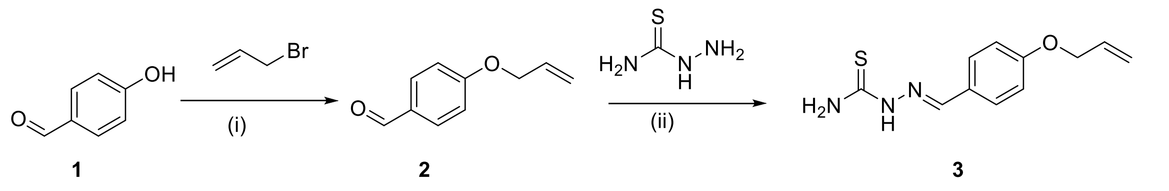

Scheme: (i) K_2CO_3, acetone, 12h, 60°C, 80%. (ii) conc. H_2SO_4 (cat), EtOH, reflux, overnight, 76%

1.1) General procedure for the synthesis of 4-(allyloxy)benzaldehyde[1,2] ***(2)*** :

At room temperature, allyl bromide (1.5 mL, 18.03 mmol) was gently added to a stirred solution of 4-hydroxybenzaldehyde (1, 2.0 g, 16.39 mmol) and potassium carbonate (6.7 g, 49.18 mmol) in acetone (20 mL) and allowed to stir for 12 hours at 60°C. After completion of the reaction as monitored by TLC, excess of acetone was evaporated under reduced pressure. The crude product was diluted with water and ethyl acetate (3 × 50 mL). Na_2SO_4 was added to combined organic layers, while excess of solvent was evaporated *in vacuo*. Pure compound **2** was obtained from column chromatography using ethyl acetate-hexane as the eluent. Colorless liquid, yield 80%. ^1H NMR (400 MHz, DMSO- d_6): δ 9.88 (s, 1H), 7.83 (d, J = 8.8 Hz, 2H), 7.01 (d, J = 8.8 Hz, 2H), 6.00-6.10 (m, 1H), 5.41-5.46 (m, 1H), 5.32-5.35 (m, 1H), 4.62 (d, J = 5.4 Hz, 2H) ppm.

1.2) General procedure for the synthesis of (E)-2-(4-(allyloxy)benzylidene)hydrazine-1-carbothioamide[2] ***(3)*** :

At room temperature, the appropriate volume of concentrated sulfuric acid was added to a stirred solution of *4-(allyloxy)benzaldehyde* (**2**, 1.5 g, 9.26 mmol) in ethanol (10 mL) and thiosemicarbazide (925 mg, 10.18 mmol). Overnight, the resultant mixtrue was allowed to reflux. The reaction mixture was cooled to room temperature after completion of the reaction, and the resulting solid was carefully washed and purified by recrystallization to give the desired compound **3.** Off white solid, yield 76%. Mpt: 160-162°C. ^1H-NMR (400 MHz, DMSO- d_6): δ 11.28 (s, 1H), 8.08 (s, 1H), 7.95 (s, 1H), 7.88 (s, 1H), 7.69 (d, J = 8.9 Hz, 2H), 6.93 (d, J = 8.9 Hz, 2H), 5.96-6.05 (m, 1H), 5.36 (d, J = 17.3 Hz, 1H), 5.24 (d, J = 10.6 Hz, 1H), 4.56 (m, 2H) ppm. ^{13}C NMR (100 MHz, DMSO- d^6): δ 178.11, 160.10, 142.66, 133.98, 129.42, 127.39, 118.04, 115.35, 68.79 ppm.

2) Spectral Data of compounds

2.1) ^1HNMR of *4-(allyloxy)benzaldehyde* ***2*** **::**

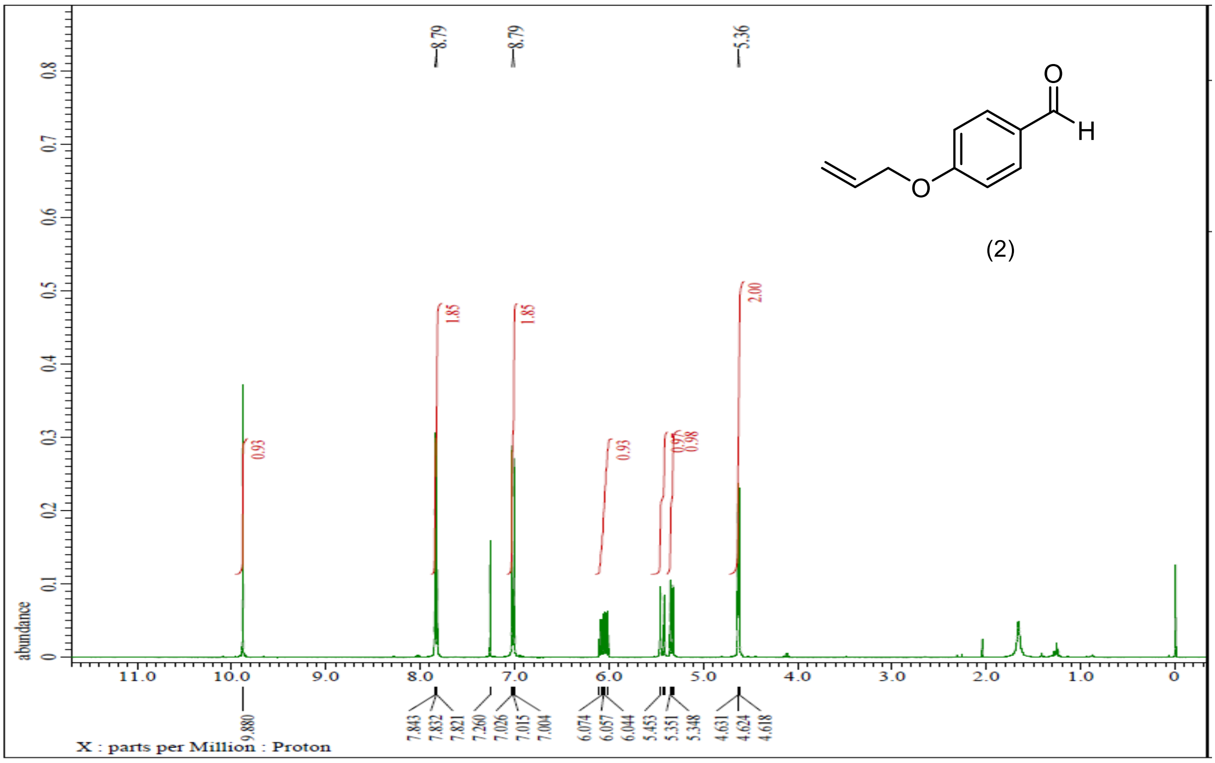

2.2) ^{1}H NMR of (E)-2-(4-(allyloxy)benzylidene)hydrazine-1-carbothioamide (3):

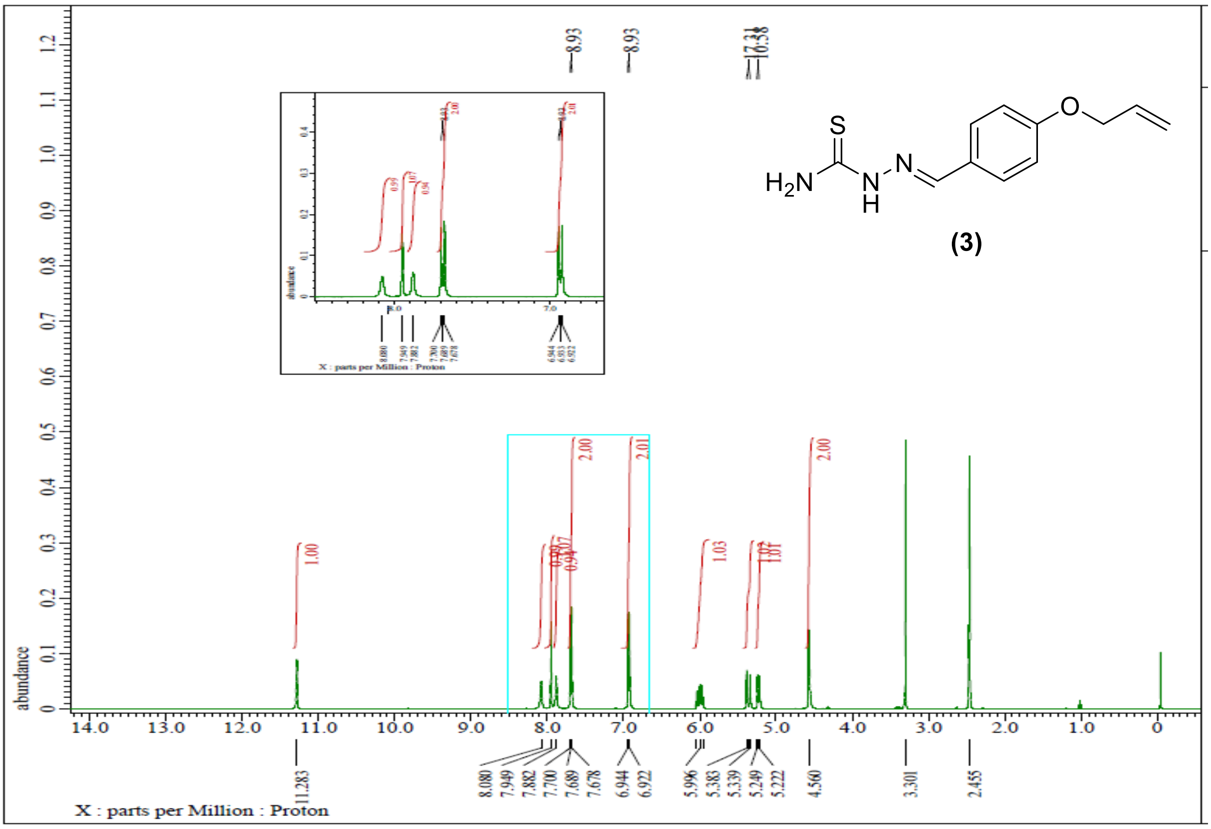

^{13}C NMR of (E)-2-(4-(allyloxy)benzylidene)hydrazine-1-carbothioamide (3):

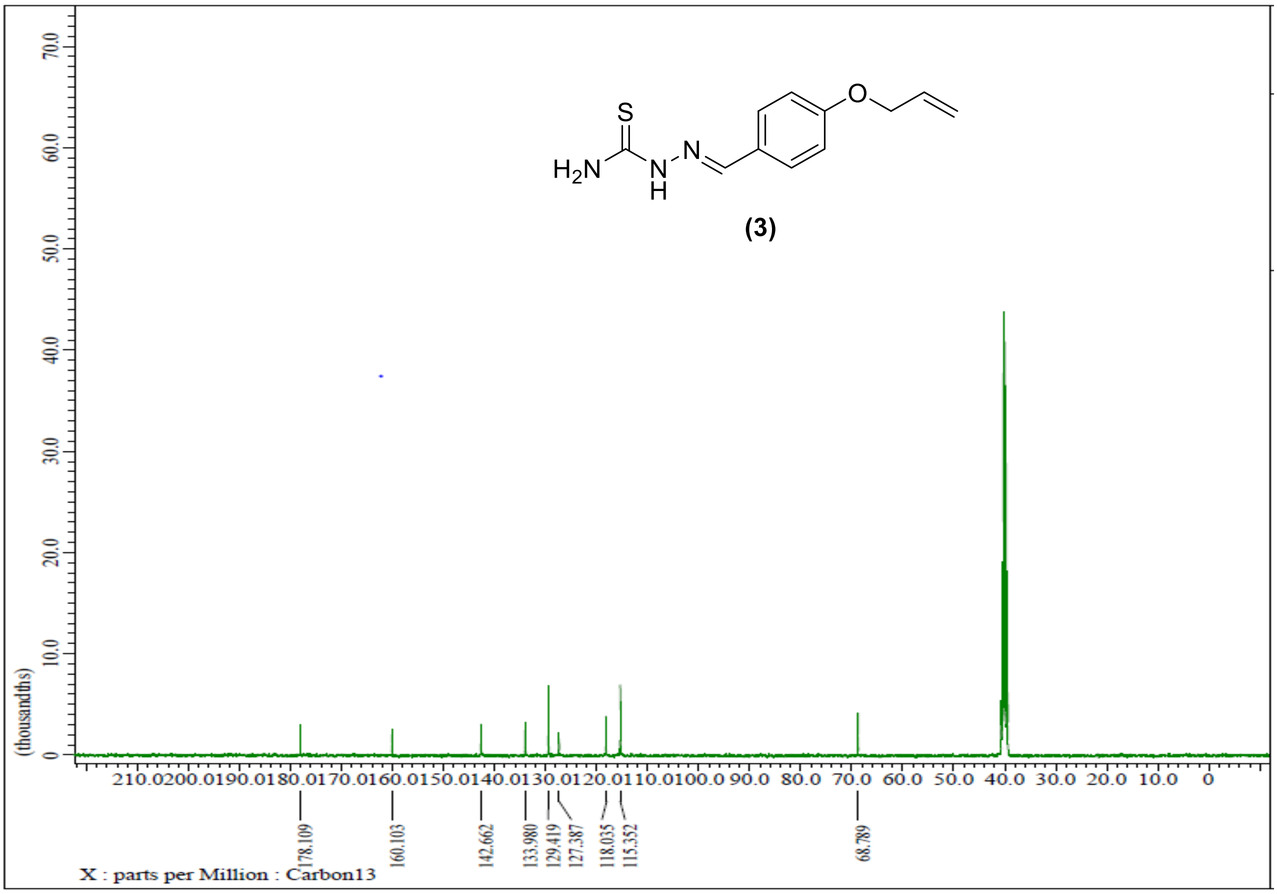

References:

1. Gu, W. and Silverman, R.B. New stable backbone linker resins for solid-phase peptide synthesis. *Org. lett.,* **2003**, *5*(4), 415-418.
2. Yi, W., Cao, R., Chen, Z., Yu, L., Wen, H., Yan, Q., Ma, L.; Song, H. Rational design and synthesis of 4-o-substituted phenylmethylenethiosemicarbazones as novel tyrosinase inhibitors. *Chem. Pharma. Bull.,* ***2010****, 58*(5), 752-754.

Supplementary Table 1: Combination Profile of NSC19723 with Rifampicin, Isoniazid, Bedaquiline or PA-824

Drug combination	MIC (nM)		FIC of drugs in combination	∑FIC[#]	Outcome
	Alone	Combination			
**NSC19723**<br>**Rifampicin**	391<br>5	12<br>2.5	0.03125<br>0.5	0.531	Indifferent
**NSC19723**<br>**Isoniazid**	391<br>400	1.5<br>200	0.003<br>0.5	0.503	Indifferent
**NSC19723**<br>**Levofloxacin**	391<br>400	48<br>200	0.125<br>0.5	0.625	Indifferent
**NSC19723**<br>**Bedaquiline**	391<br>400	97<br>100	0.25<br>0.25	0.5	Synergistic
**NSC19723**<br>**PA-824**	391<br>400	97<br>100	0.25<br>0.25	0.5	Synergistic

#FICI value ≤0.5 indicates synergistic activity, FICI of ≥4.0 indicates antagonistic activity, and values in between ≤ 4.0 and ≥0.5 indicate indifferent interaction. The data shown in obtained from two experiments performed in duplicates.

Supplementary Table 2: Combination Profile of Thiacetazone with Rifampicin, Isoniazid, Bedaquiline or PA-824

Drug combination	MIC (nM)		FIC of drugs in combination	∑FIC#	Outcome
	Alone	Combinatio n			
**Thiacetazone**<br>**Rifampicin**	1565<br>5	195<br>2.5	0.125<br>0.5	0.625	Indifferent
**Thiacetazone**<br>**Isoniazid**	1565<br>400	391<br>200	0.25<br>0.5	0.75	Indifferent
**Thiacetazone**<br>**Levofloxacin**	1565<br>400	391<br>200	0.25<br>0.5	0.75	Indifferent
**Thiacetazone**<br>**Bedaquiline**	1565<br>400	391<br>100	0.25<br>0.25	0.5	Synergistic
**Thiacetazone**<br>**PA-824**	1565<br>400	391<br>100	0.25<br>0.25	0.5	Synergistic

#FICI value ≤0.5 indicates synergistic activity, FICI of ≥4.0 indicates antagonistic activity, and values in between ≤ 4.0 and ≥0.5 indicate indifferent interaction. The data shown in obtained from two experiments performed in duplicates.
